# No evidence of sex-ratio meiotic drive associated with the *X*-linked *In(1)Be* inversion in *Drosophila melanogaster*

**DOI:** 10.1093/g3journal/jkag181

**Published:** 2026-07-24

**Authors:** Madison E An, David J Begun

**Affiliations:** Department of Evolution and Ecology, University of California, Davis, CA 95616, United States; Department of Evolution and Ecology, University of California, Davis, CA 95616, United States

**Keywords:** meiotic drive, selfish genetic element, *X*-linked inversion, *D. melanogaster*

## Abstract

Sex-ratio (SR) *X* chromosomes are selfish genetic elements that increase the rate of *X* chromosome transmission in species with a heterogametic sex, violating Mendel's first law of segregation. In particular, heterogametic males carrying SR *X* chromosomes produce a greater number of *X*-bearing sperm than *Y*-bearing sperm and thus produce a greater number of female than male offspring. SR *X* chromosomes have been observed in several taxa, including multiple *Drosophila* species. However, no convincing examples of a strong SR *X* chromosome have been identified in *Drosophila melanogaster*. There have been few reports of weak SR *X* chromosomes segregating in *D. melanogaster*, including speculation of the *X*-linked inversion *In(1)Be* being associated with an SR phenotype. We sought to investigate if the *In(1)Be* inversion is associated with an SR by measuring the offspring SR in genetically controlled experiments. In our experiments, we found no significant differences in SR between karyotype or across our generated lines, suggesting that in the genetic background we used, there is no SR phenotype associated with the *In(1)Be* inversion.

## Teaser

Sex-ratio *X* chromosomes are classic examples of selfish genetic elements. Some investigations have reported sex-ratio *X* chromosomes in *D. melanogaster* associated with an *X*-linked inversion, *In(1)Be*. However, in the authors’ genetically controlled experiments they find no evidence of a sex ratio correlated with *In(1)Be*.

## Introduction

In species with heterogametic males (e.g. XY males in *Drosophila*), Mendel's first law dictates that males should sire equal numbers of male and female offspring. However, this expectation can be subverted by selfish genetic elements, known as sex-ratio (SR) *X* chromosomes (Sandler and Novitski 1957; Jaenike 2001), which have been observed in several taxa, including multiple Drosophila species (Curtsinger and Feldman 1980; Jaenike 1996; Tao et al. 2007; Helleu et al. 2015). Heterogametic males carrying an SR chromosome transmit a greater number of X-bearing than Y-bearing sperm, and thus sire a greater number of female than male offspring (Jaenike 2001). In the strongest SR systems, males may sire only daughters, though the strength of the SR phenotype may vary widely (Jaenike 1996; Tao et al. 2007; Helleu et al. 2015). SR systems favor transmission of X-bearing sperm by reducing the viability of Y-bearing sperm postmeiotically, though the molecular mechanisms underlying these sperm development defects remain poorly understood.

SR chromosomes represent a classic case of countervailing selection pressure at the gametic and zygotic levels. SR chromosomes have an advantage at the gametic level due their transmission advantage but are also associated with reductions in numbers of viable sperm, which may reduce male fitness. The population genetics of *X* chromosome drive are complex and depend on effects at different levels, including the strength of transmission distortion, the effects on various male fertility phenotypes or other traits, and possible effects of SR chromosomes on females. If left unchecked, SR chromosomes may rapidly spread to fixation and drive a population to extinction (Hamilton 1967). If the gametic selective advantage outweighs any negative zygotic fitness effects, SR chromosomes will invade a population. Indeed, multiple *Drosophila* species segregate SR chromosomes that decrease male fertility (Wu 1983; Jaenike 1996; Atlan et al. 2004). The deleterious nature of sex chromosome drive on organismal fitness components and *Y* chromosome transmission favors the evolution of suppressors which can reduce or eliminate the drive phenotype. These suppressors can arise anywhere in the genome, including autosomes, and have been reported in multiple *Drosophila* species (Carvalho et al. 1997; Jaenike 2001; Atlan et al. 2003; Tao et al. 2007; Lyth et al. 2025). However, drivers which can evade or overcome suppressors will again increase their transmission. This intragenomic conflict may create an evolutionary arms race, which could explain how *X* chromosome drive is maintained in a species (Sandler and Novitski 1957; Curtsinger and Feldman 1980; Vaz and Carvalho 2004).

While SR *X* chromosomes have been characterized in multiple species, no convincing examples of strong SR *X* chromosomes have been identified in the classic genetic model system, *Drosophila melanogaster*, which has limited functional investigation of these selfish chromosomes. However, some investigations have reported weak SR *X* chromosomes segregating in the species. Given the genetic tools available for *D. melanogaster*, confirmation of natural SR *X* chromosomes in the species could be a great benefit in better understanding the development genetics of such selfish genetic elements.

Two papers, Curtsinger (1984) and Reed et al. (2005), used quantitative genetic approaches to detect possible weak SR *X* chromosomes. These approaches attempted to partition transmission bias of *X* chromosomes through males into viability and segregation components. However, such approaches require several assumptions, and deviations from these assumptions may be difficult to estimate. Curtsinger found little evidence of segregation bias among 18 wild *X* chromosome lines originating from Texas. Of these 18 lines, only three showed statistical evidence of segregation effects, all of which were small and less-than-expected transmission of the *X* relative to Mendelian expectation (which is not supportive of the presence of SR X chromosomes). Reed et al. used a collection of isofemale lines from multiple geographic locations and, in an F2 design, reported evidence of relatively weak *X*-biased transmission in males from six of the 49 assayed lines. Viability effects were modeled using simplifying assumptions but were empirically estimated for only about half the lines and only four of the six significant lines. Notably, point estimates of proportion of female offspring were greater than 0.5 for 40 of the 49 assayed lines, consistent with frequent, weak SR phenotypes but also consistent with uncontrolled phenotypic variation in the experiment that generally resulted in slight female-biased offspring counts.

Corbett-Detig and Hartl (2012) carried out a population genomic analysis of *D. melanogaster* inversion polymorphism. As part of their work, they speculated, based on earlier literature reporting associations between SR X chromosomes and X-linked inversions in other *Drosophila* species (Jaenike 2001; Dyer et al. 2007), and on the mapping results from Reed et al., that an *X*-linked *D. melanogaster* inversion, *In(1)Be*, was associated with an SR phenotype. They tested this idea by crossing *In(1)Be* and *Standard* lines to isofemale lines and then examining the transmission of *X* and *Y* chromosomes by the resulting male offspring. While there were small significant deviations from Mendelian segregation for three of the four crosses using the inversion, these deviations were dependent on the specific genotype of the male parent of the experiment animals, suggesting significant effects of genetic background. Moreover, the number of tested males from each inversion genotype were small, and the control crosses also showed consistent though nonsignificant excesses of female offspring sired by *St* (i.e. noninversion) fathers. Overall, these reports provide weak evidence in support of the hypothesis that *D. melanogaster* segregates SR *X* chromosomes.

In the experiments described here we set out to create a collection of male genotypes that carried either *In(1)Be* or *St X* chromosomes but had identical *Y* chromosomes and autosomes. Thus, the only source of variation in these experiments is the *X* chromosome genotypes. We then measured for this collection of genotypes the SR of their offspring when mated to a single inbred tester female genotype. The experimental design enables a more genetically controlled test of the hypothesis of Corbett-Detig and Hartl that *In(1)Be* is a weak SR distorter.

## Methods

### Fly maintenance and stocks used

Flies were maintained in bottles with standard yeast-cornmeal-agar food and kept at 25C. Bottles were transferred weekly. Flies were aged to 2 to 3 days old before being used in SR measuring crosses, with a maximum of five flies per vial.

### Identifying *In(1)Be* and *St* X chromosome lines

We molecularly screened a collection of isofemale lines from Panama for *In(1)Be* vs *St* status, as previous work suggested the inversion was common in this population sample (Cridland et al. 2023), exhibiting a frequency of about 15%. This was accomplished following the PCR screen from Corbett-Detig and Hartl (2012). Genomic DNA was extracted from flies from each isofemale line (Cridland et al. 2023). Molecular karyotyping was performed using *In(1)Be*- and *St*-specific PCR primer pairs from Corbett-Detig and Hartl (2012).

The *In(1)Be* inversion containing lines from Panama were PC12 and PC65. We also used a single line from the DPGP2, GA191, which had previously been characterized bioinformatically as containing *In(1)Be* (Pool et al. 2012). This line was validated as *In(1)Be* by PCR as above. The *St X* chromosome lines were all from the Panama sample: PC82, PC136, PC161, and PC192.

### Generating attached-X lines

We used a *C(1)DX* line from Bloomington Drosophila Stock Center (#11298) to generate a stable attached-X line for each experimental (i.e. *In(1)Be*) and control (i.e. *St*) strain. A series of three crosses were used to generate X^X stocks derived from each *In(1)Be* and *St* line. The *C(1)DX* stock used is maintained as *X^X/Y* females and *X/Y* males and carries *bw* and *st* on chromosomes 2 and 3, respectively. Flies containing both *bw* and *st* markers have white eyes, allowing us to isogenize chromosomes 2 and 3 by selecting for white eyed males from the second cross. The crosses were as follows:

Cross 1: single X*/*Y*; *+; +* ♂ x *C(1)DX*,*y*,*f/Y; bw; st* ♀

↓

Cross 2: single X*/Y; +/b*w*; +/*st* ♂ x *C(1)DX,y,f/Y; bw; st* ♀

↓

Cross 3: single X*/Y; bw; st ♂ (white-eyed) x *C(1)DX,y,f/Y; bw; st* ♀

↓

stable *X^X* stock

*In(1)Be or *St* X chromosome from original line

These final attached-*X* lines were all subjected to the PCR karyotype screen again before being used for estimation of the SR phenotype. All lines survived this validation step. The resulting lines have the same Y and autosome genotypes, varying only in the X chromosome.

### Measuring SR

Virgin *In(1)Be* or *St* males from attached-*X* stocks and virgin RAL517 females were collected and aged for 2 to 3 d before being used in crosses. For each line we placed four males in a vial with four RAL517 females for either 24 or 48 h before removal. Vials were kept at 25C. The numbers of female and male offspring per vial were counted 14 days after the crosses were set up. The SR is estimated as the ratio of females to males. We imposed a minimum of 20 offspring cutoff for a vial to contribute to the SR estimate.

### Statistical analysis

The data were analyzed using R v. 4.3.1. We used a binomial generalized linear mixed model implemented in glmer (lme4 package) to estimate the effect of X chromosome status (*St* or *In(1)Be*) on offspring SR, here defined as the ratio of females to males. X chromosome karyotype (*St* or *In(1)Be*) was a fixed effect and individual lines were included as a random effect. The binomial GLMM tested whether offspring SRs significantly differed between *St/Y* and *In(1)Be/Y* males by comparing the log-odds of an offspring being female between the two groups. We also tested for heterogeneity of offspring SR between *St/Y* and *In(1)Be/Y* males and among individual lines by performing a nested analysis of variance (ANOVA) using the estimated SRs. In the nested ANOVA, individual lines were nested within X chromosome karyotype.

## Results and discussion

To investigate the proposed link between the *In(1)Be X* chromosome inversion and an SR phenotype we generated three attached-*X* lines for which males carried *In(1)Be* and four attached-*X* lines for which males carried a *Standard X* karyotype. Males from all seven lines carried the same *Y* chromosome, second chromosome, and third chromosome genotypes, all of which derive from the original attached-*X* stock.

After filtering for a minimum of 20 offspring per vial, the four experimental *St/Y* strains yielded a sample size of 108 *St/Y* males across 27 vials. The three experimental *In(1)Be/Y* strains yielded a sample size of 84 *In(1)Be/Y* males across 21 vials. To increase the sample size of vials containing at least 20 offspring, we then carried out a second experiment where we allowed flies to mate for 48 h, which after filtering for at least 20 offspring per vial yielded 180 *St/Y* males across 45 vials and 164 *In(1)Be/Y* males across 41 vials. A total of 288 *In(1)Be/*Y males and 248 *St/Y* males were tested across the two experiments. We found no statistical differences between the 24-h mating and 48-h mating experiments and so they were pooled in the analyses presented below.

**Fig. 1.**
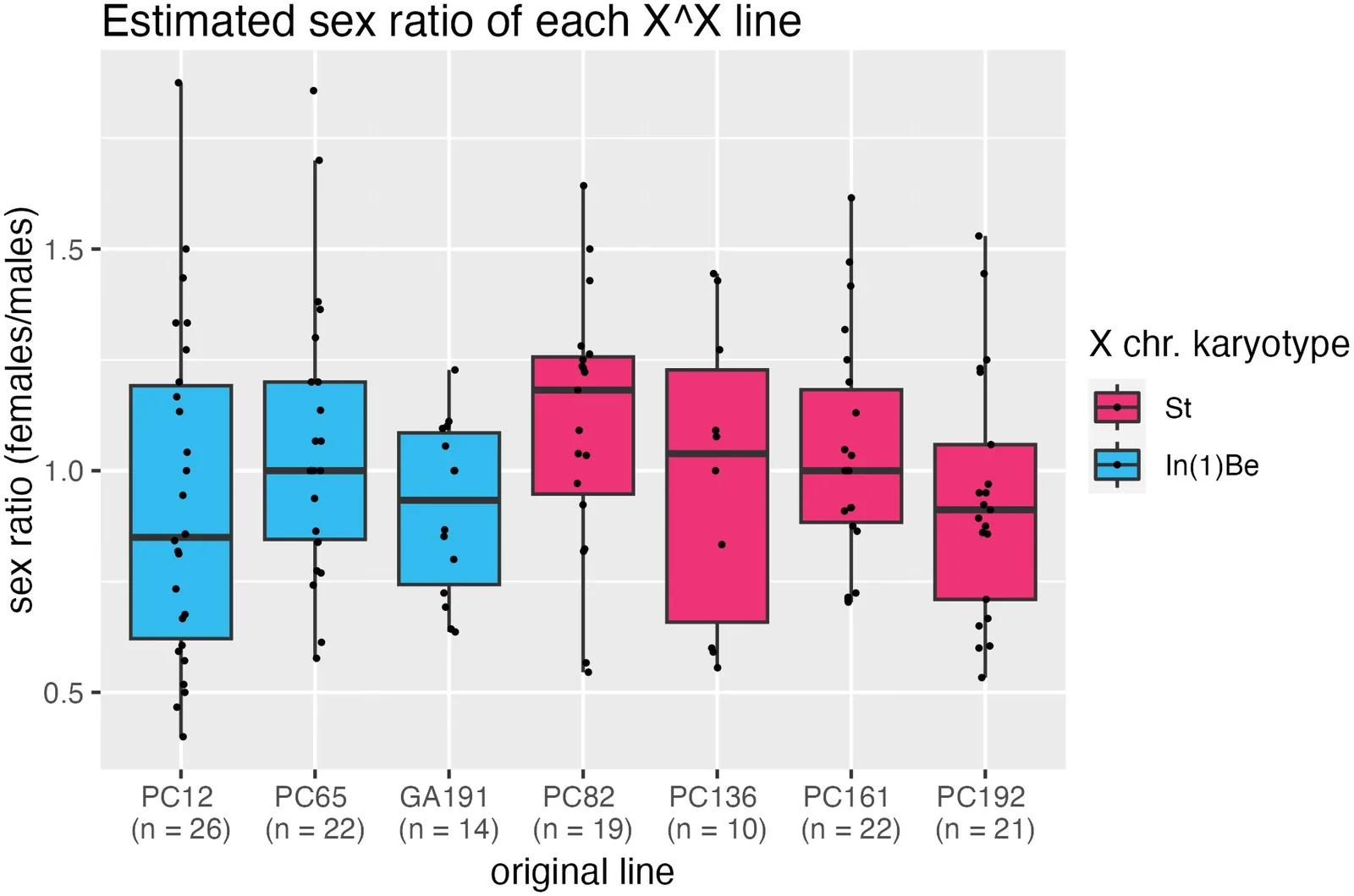
Estimated sex ratios for individual X^X lines. The original line used to generate the X^X stock is indicated on the *x*-axis with sample size (number of vials counted) listed below. The left three groups are *In(1)Be* lines and the right four groups are *St* lines Sex ratio is measured as ratio of females to males.

**Fig. 2.**
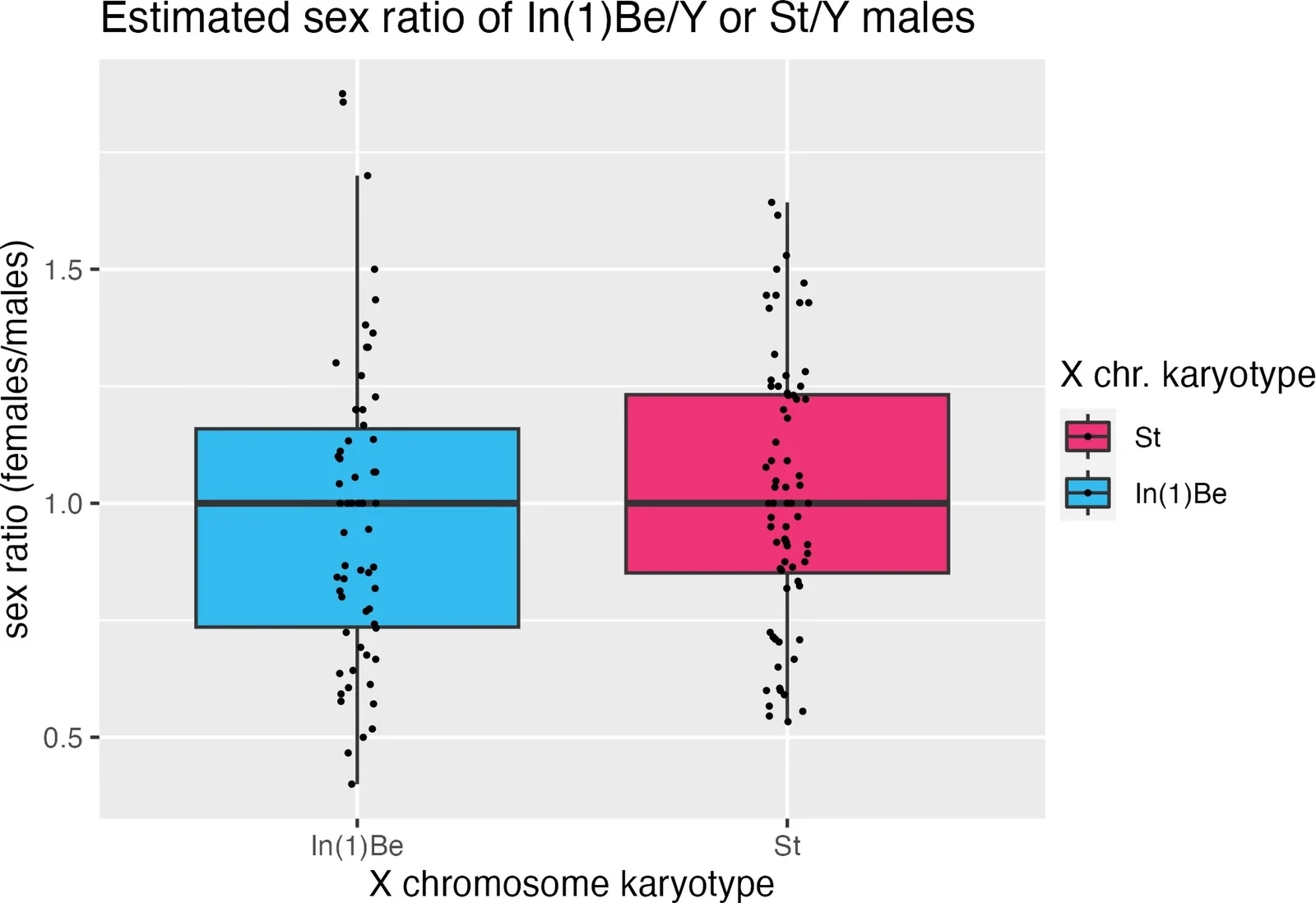
Estimated sex ratios for *St* (right) and *In(1)Be* (left) males. Sex ratio is measured as ratio of females to males.

The median offspring SRs for all lines individually ≈1 indicating a roughly equal number of female and male offspring across all lines (Fig. 1). In a nested ANOVA directly comparing the SRs we find no significant heterogeneity among lines (*P* = 0.289) (Table 1). When pooling the data for individual lines into *In(1)Be/Y* or *St/Y* males, we observed a total of 1,239 females and 1,366 males from *In(1)Be/Y* males and 1,464 females and 1,499 males from *St/Y* males. For both *In(1)Be/Y* and *St/Y* pooled data the median offspring SR = 1; the nested ANOVA indicates no significant difference in SRs for the two X chromosome karyotypes (*P* = 0.396) (Fig. 2 and Table 1). These results support the null hypotheses of no differences in SR between *In(1)Be/Y* males and *St/Y* males as well as between individual lines. Additionally, the SRs do not deviate from Mendelian expectations.

**Table 1.** Nested ANOVA table for sex-ratio comparison between *X* chromosome karyotype and individual lines.

Source	*df*	Sum sq	Mean sq	*F* value	*P* value
*X* chr. karyotype	1	0.066	0.06603	0.726	0.396
Line	5	0.569	0.11383	1.252	0.289
Residuals	127	11.551	0.09095		

To further analyze the ratio of female to male offspring produced by *In(1)Be/Y* vs *St/Y* males, we used a binomial GLMM comparing the log-odds of an offspring being female. Using our 24-h mating data, we see no significant difference in the proportion of offspring being female between *In(1)Be/Y* and *St/Y* males (*P* = 0.609). The 48-h mating data yielded similar results finding no significant difference between *In(1)Be/Y* and *St/Y* males (*P* = 0.204). The combined 24- and 48-h mating data also revealed no indication of an SR phenotype (*P* = 0.241).

We conclude that our experiments showed no good evidence of an SR phenotype associated with the *In(1)Be* inversion, which does not support the hypothesis of Corbett-Detig and Hartl. While we did not estimate viability effects, the fact that we observed no heterogeneity among lines or karyotypes and no deviations from Mendelian expectation, strongly suggests that *X*-linked viability effects are very unlikely to have influenced our results. While our experiment is well controlled genetically, our conclusion that there is no SR phenotype associated with the *In(1)Be* inversion, is limited to the particular genetic background used in our experiment, which differs from those used by Corbett-Detig and Hartl. It remains possible that this inversion would exhibit weak SR drive in other genetic contexts.

## Data Availability

The authors affirm that all data necessary for confirming the conclusions of the article are present within the article, figures, and tables.
